# Who makes it all the way? Participants vs. decliners, and completers vs. drop-outs, in a 6-month exercise trial during cancer treatment. Results from the Phys-Can RCT

**DOI:** 10.1007/s00520-021-06576-0

**Published:** 2021-09-28

**Authors:** Emelie Strandberg, Christopher Bean, Karianne Vassbakk-Svindland, Hannah L. Brooke, Katarina Sjövall, Sussanne Börjeson, Sveinung Berntsen, Karin Nordin, Ingrid Demmelmaier

**Affiliations:** 1 Department of Public Health and Caring Sciences, Uppsala University, Uppsala, Sweden; 2 Department of Oncology and Skåne University Hospital, Department of Oncology, Lund University, Lund, Sweden; 3 Department of Oncology and Department of Health, Medicine and Caring Sciences, Linköping University, Linköping, Sweden; 4 Department of Sport Science and Physical Education, University of Agder, Kristiansand, Norway

**Keywords:** Physical activity, Oncology, Recruitment, Behavior, Health psychology, Attrition

## Abstract

**Purpose:**

To compare sociodemographic, health- and exercise-related characteristics of participants *vs.* decliners, and completers *vs.* drop-outs, in an exercise intervention trial during cancer treatment.

**Methods:**

Patients with newly diagnosed breast, prostate, or colorectal cancer were invited to participate in a 6-month exercise intervention. Background data for all respondents (*n* = 2051) were collected at baseline by questionnaire and medical records. Additional data were collected using an extended questionnaire, physical activity monitors, and fitness testing for trial participants (*n* = 577). Moreover, a sub-group of decliners (*n* = 436) consented to additional data collection by an extended questionnaire . Data were analyzed for between-group differences using independent *t*-tests and chi^2^-tests.

**Results:**

Trial participants were younger (59 ± 12yrs *vs.* 64 ± 11yrs, *p* < .001), more likely to be women (80% *vs.* 75%, *p* = .012), and scheduled for chemotherapy treatment (54% *vs.* 34%, *p* < .001), compared to decliners (*n* = 1391). A greater proportion had university education (60% *vs* 40%, *p* < .001), reported higher anxiety and fatigue, higher exercise self-efficacy and outcome expectations, and less kinesiophobia at baseline compared to decliners. A greater proportion of trial participants were classified as ‘not physically active’ at baseline; however, within the group who participated, being “physically active” at baseline was associated with trial completion. Completers (*n* = 410) also reported less kinesiophobia than drop-outs (*n* = 167).

**Conclusion:**

The recruitment procedures used in comprehensive oncology exercise trials should specifically address barriers for participation among men, patients without university education and older patients. Individualized efforts should be made to enroll patients with low exercise self-efficacy and low outcome expectations of exercise. To retain participants in an ongoing exercise intervention, extra support may be needed for patients with kinesiophobia and those lacking health-enhancing exercise habits at baseline.

## Background

Exercise, defined as planned, structured physical activity with the aim to improve or sustain physical function and/or fitness [7], is generally safe and beneficial during and after cancer treatment [6]. Reviews of exercise trials including cancer survivors report positive effects on physical fitness [17, 37], health-related quality of life [12], and cancer-related fatigue [26]. Furthermore, exercise during and after treatment may increase chemotherapy completion rates [8, 39] and reduce the risk of cancer mortality [22, 24]. Based on this evidence, international guidelines for cancer survivors recommend three weekly sessions of at least moderate-intensity endurance training, and/or two weekly sessions of resistance training, to reduce frequently occurring secondary health problems due to cancer and cancer treatment [6].

Recruitment rates of eligible patients to exercise trials vary between 9.5 and 44% [8, 16, 18, 34, 38, 40], which poses a threat to external validity. Most exercise trials in cancer patients have been performed in highly selected samples with more favorable sociodemographic profiles than the intended target population. For example, participants are more likely to have a university education [18, 40] and higher levels of social support [40] compared to non-participants. In addition, participants are younger [13, 41], less physically active at baseline [40], and report lower levels of psychological distress [18] and cancer-related fatigue [13, 40]. Additional modifiable variables such as fear of movement (kinesiophobia) [19], exercise self-efficacy [2], and outcome expectations [1] may also differ between trial participants and non-participants, but these factors have typically received less attention in the literature.

Although differences related to patient characteristics between those who participate versus those who do not have been investigated in a small number of studies [13, 18, 40, 41], more knowledge regarding modifiable and non-modifiable variables that are likely to influence trial participation would contribute to a better understanding of how to approach patients who may be reluctant to partake in exercise programs during oncological treatment. In addition, withdrawal from exercise trials is common among cancer populations [36]. However, differences in characteristics between completers and drop-outs have not been previously explored in patients with cancer. Identifying such differences may provide a better understanding of how to motivate participants who are likely to drop-out from exercise programs. Altogether, increasing knowledge about differences in characteristics between participants and decliners, as well as between completers and drop-outs, is important to better understand the selection process during recruitment and enrollment in order to develop strategies to improve participation and enhance generalizability. The present study aimed to compare sociodemographic, health-related, and exercise-related characteristics between participants and those who declined participation in a randomized controlled exercise trial performed during cancer treatment. An additional aim was to compare the same characteristics between those who completed the 6-month exercise intervention and those who withdrew before the end of the intervention.

## Methods

### The Phys-Can randomized controlled trial

This study used data from the Physical Training and Cancer (Phys-Can) intervention study [3, 9]. The primary aim of the Phys-Can RCT was to compare the effects of low-to-moderate (LMI) *vs.* high-intensity (HI) exercise, with or without additional behavior change support (goal-setting, self-monitoring, action planning, review of goal-setting, and problem solving), on cancer-related fatigue in patients undergoing cancer treatment. The participants were randomized into one of four groups: two HI, and two LMI; one of each intensity level group receiving additional behavioral change support. The supervised group-based resistance training was performed at public gyms twice per week over a 6-month period. The LMI groups performed 3 sets, alternating between 12 repetitions (once a week) and 20 repetitions (once a week) with a corresponding training load of 50% of 6 and 10 RM (repetitions maximum), respectively. The HI groups performed 3 sets alternating between 6 RM (once a week) and 10 RM (once a week) until failure in the third set. Endurance training was home-based and the LMI groups performed 150 min/week at 40–50% of individual HRR (heart rate reserve), mainly walking or cycling. The HI groups performed 5 × 2-min intervals of running or cycling at 80–90% of HRR twice a week, with progression from 5 to 10 intervals over the 6-month training period.

Participants in the Phys-Can RCT were recruited from three Swedish university hospitals between 2015 and 2018. Eligible participants were adults (age > 18 years), literate in Swedish, and recently diagnosed with curable breast (BC; women only), prostate (PC), or colorectal cancer (CRC), and scheduled to begin (neo-)adjuvant cancer treatment. Exclusion criteria were stage IIIb-IV BC, inability to perform basic activities of daily living, cognitive disorders, disabling conditions that might contraindicate HI exercise, treatment for an additional ongoing malignant disease or orthopedic conditions, BMI < 18.5 kg/m^2^, or pregnancy. A research nurse/assistant provided oral and written information to eligible participants prior to start of the treatment.

### Participants and data collection

**Fig. 1:**
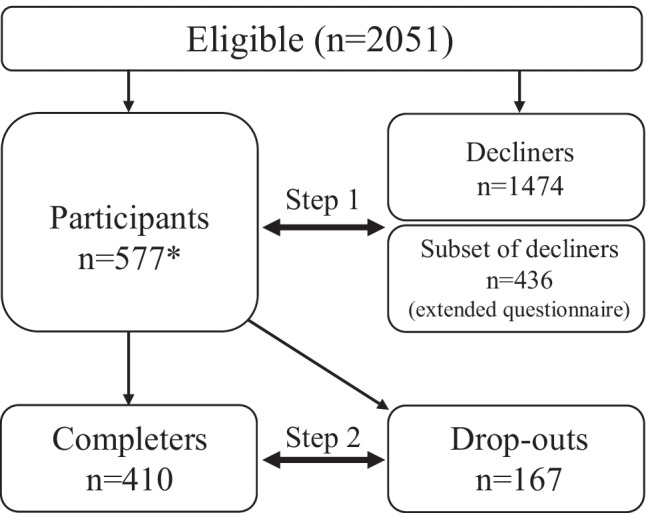
Study flowchart and steps in analysis. *A total of 23 participants withdrew before randomization, resulting in *n* = 577

Background data for all patients eligible for the RCT (*n* = 2051) were collected at baseline by questionnaire (age, sex) and medical records (cancer diagnosis and treatment modality: chemotherapy or not). Six hundred out of 2051 invited eligible patients agreed to participate in the RCT; however, twenty-three participants withdrew from the study before randomization resulting in 577 RCT participants. Additional data were collected by an extended questionnaire, and by using a physical activity monitor and fitness testing at baseline for RCT participants (*n* = 577). Moreover,  a sub-group of decliners (*n* *=* 436)  consented to additional data collection by an extended questionnaire. The extended questionnaire included sociodemographic variables (education, living situation) and a number of variables related to health and exercise as described below. Among the 577 RCT participants, we identified one group who completed the intervention; “completers” (*n* = 410) and one group who withdrew before the end of the intervention; “drop-outs” (*n* = 167) (see Fig. 1).

*Treatment modality*, chemotherapy treatment or not, was assessed by questionnaire for decliners, and by medical records for trial participants.

*Comorbidity* was assessed with a standard list of conditions including heart disease, respiratory disease, arthritis, diabetes, diseases of the digestive tract, migraine, psychiatric problems, and diseases of the central nervous system.

*Anxiety and depression* were assessed with the 14-item Hospital Anxiety and Depression Scale (HADS) [43]. It includes two subscales with 7 items on anxiety and 7 items on depression. Responses are given on a 0–3 scale, resulting in a sum score of 0–21 for each subscale, with higher scores indicating more anxiety or depression.

*Cancer-related fatigue* was assessed with the Multidimensional Fatigue Inventory (MFI) [35]. The 20-item MFI includes five subscales: general fatigue, physical fatigue, reduced activity, reduced motivation, and mental fatigue. Responses are given on a 1–5 scale, resulting in a sum score of 4–20 for each subscale, with higher scores indicating more fatigue.

*Exercise stage* at the time of invitation to the study was assessed with the Exercise Stage Assessment Instrument (ESAI) [28], based on the Transtheoretical Model Stages of Change [32]. Participants responded to two questions: “Which one of the statements below is most accurate to describe your current level of *endurance training*?” and “Which one of the statements below is most accurate to describe your current level of *resistance training*?.” Responses are given on a 5-point scale representing different stages of change; (1) “Not very physically active and I do not intend to become more physically active during the next 6 months” (pre-contemplation stage); (2) “Not very physically active but I have considered increasing my activity during the next 6 months” (contemplation stage); (3) “Not very physically active but determined to increase my activity during the next 6 months” (preparation stage); (4) “Physically active, but only during the previous 6 months” (action stage); and (5) “Physically active and I have been so for more than 6 months” (maintenance stage). For analysis, the answers were dichotomized into “not physically active” and “physically active.” The “not physically active” category included exercise stages 1, 2, and 3 (“pre-contemplation,” “contemplation,” and “preparation”), while the “physically active” category included exercise stages 4 and 5 (“action” and “maintenance”).

O*utcome expectations* were assessed with three study-specific questions: “How certain are you that exercise…” (1) “is healthy for you,” (2) “can reduce symptoms from cancer treatment,” (3) “can reduce the risk of relapse?” Responses for each question are given on a 0–10 scale with 0 = “not at all certain” to 10 = “extremely certain.” These items have been adapted from a previous exercise intervention study in people with rheumatoid arthritis [29].

*Exercise self-efficacy* was assessed with the Exercise Barriers Self-efficacy Scale (EBSS) [33], which includes 9 barrier items encompassing lack of discipline, time, interest, inspiration, and support for exercise, as well as fatigue, nausea, bad weather, and not prioritizing exercise. Responses to each barrier are given on a 0–10 scale with 0 = “not at all certain” to 10 = “extremely certain,” resulting in a sum score of 0–90, with higher scores indicating higher exercise self-efficacy.

*Kinesiophobia* (fear of movement) was assessed using a 14-item Swedish version of the Tampa Scale for Kinesiophobia (TSK-SV-14), which is a shortened and translated version of the original TSK [23]. The questionnaire asks respondents to rate their level of agreement to 14 statements about their attitudes to physical activity. Each statement (e.g., “I cannot do the same things as others because there is too much risk of being injured”) is answered with a 4-point Likert scale (1 = strongly disagree, 2 = disagree, 3 = agree, 4 = strongly agree). Four items (3, 6, 9, and 13) are reverse-worded statements. Total scores can range from 14 to 56, with higher scores indicating greater fear of movement. The TSK-SV-14 is a shortened version of the previously validated TSK-SV-17 [21].

*Cardiorespiratory fitness* was assessed for those who accepted participation in the exercise trial (*n* = 577) and was measured as maximal oxygen uptake (VO_2_max [mL/kg/min]) using a modified Balke-protocol as previously described in detail [4]. Briefly, participants started walking or running at 4 km/h with an incline of 2% on a treadmill. The inclination was then increased by 1% every minute until reaching 12%, from which the speed increased 0.5 km/h per minute until exhaustion. Tests were accepted if two out of three criteria were fulfilled: (1) tester judged the test as maximal, (2) Borg RPE rating ≥ 17 [5], and (3) respiratory exchange ratio (RER) ≥ 1.1 [4].

*Moderate-to-vigorous intensity physical activity* was assessed using SenseWear Armband mini (BodyMedia Inc, Pittsburgh, PA, USA) as previously described [9]. Mean time (minutes) in moderate-to-vigorous intensity physical activity (MVPV) per 24 h was considered that of at least 3 METs according to SenseWear algorithms [11].

### Ethical approval

The Phys-Can RCT was approved by the Regional Ethical Review Board in Uppsala, Sweden (Dnr 2014/249), and registered in ClinicalTrials.gov (ClinicalTrials.gov.Id.NCT02473003). The study was conducted in accordance with the Declaration of Helsinki, and written informed consent was obtained from the participants before inclusion.

### Statistical analyses

Descriptive characteristics are presented as mean and standard deviation (SD) for continuous variables and proportions as number (*n*) and percentage (%) for categorical variables. Differences in means and proportions were analyzed by independent-samples *t*-tests and chi^2^-tests, respectively. Statistical analyses were performed with SPSS statistical software version 26 (SPSS Inc., Chicago, IL, USA).

## Results

The recruitment process and the steps in the analysis are depicted in Fig. 1. In total, six hundred (29%) out of 2051 invited eligible patients agreed to participate in the RCT. Twenty-three participants withdrew from the study before randomization. A total of 577 participants were randomized and were scheduled to initiate the training program. Of these, 167 (29%) participants withdrew prior to completion. The majority of withdrawals occurred before (*n* = 13; 8%) or during the first 4 weeks (*n* = 95; 57%) of the intervention (Table 1).

**Table 1:** Time point for dropping out from the RCT

	*n*	%
Before introduction	13	8
During introduction (weeks 1–4)	95	57
Month 2	6	4
Month 3	9	5
Month 4	11	7
Month 5	18	11
Month 6	15	9
Total	167	

### Step 1: Participants vs. decliners

The participants were younger; mean (SD) age 59 years (12) compared to the decliners; 64 years (11), *p* < 0.001. Among participants, compared to the decliners, there was a greater proportion of women (80% vs 75%, *p* = 0.012), patients diagnosed with breast cancer (79% vs 71%, *p* = 0.001), patients scheduled to undergo chemotherapy treatment (54% vs 34%, *p* < 0.001), and people with a university education (60% vs 40%, *p* < 0.001) (Table 2). Participants also reported higher levels of anxiety, more cancer-related fatigue (in four out of five subscales), higher exercise self-efficacy, and less kinesiophobia compared to the decliners (Table 2). A greater proportion of participants were classified as “not physically active” (i.e., the “pre-contemplation,” “contemplation,” or “preparation” stages according to the ESAI questionnaire). In addition, participants had higher outcome expectations of exercise regarding improved health and symptom reduction in comparison to decliners. No differences between the participants and decliners were detected regarding living situation or comorbidity.

**Table 2:** Comparison of participants in the exercise trial vs decliners, based on baseline data. *N* vary due to missing data and different methods of data collection (age, sex, diagnosis, and treatment vs remaining variables)

	Participants	Decliners		Decliners	
	*n* (%)^a^	Mean ± SD	*n* (%)^a^	Mean ± SD	*P-value*
**Age**, years	577	59 ± 12	1391	64 ± 11	< 0.001
**Sex**					
Men	112 (20)		351 (25)		0.012
Women	465 (80)		1069 (75)		
**Living situation**					
Living with partner	431 (78)		212 (77)		0.854
Living without partner	122 (22)		62 (23)		
**Education level**					
University	336 (60)		111 (40)		< 0.001
Not university	222 (40)		163 (60)		
**Diagnosis**					
Breast cancer	457 (79)		1044 (71)		0.001
Prostate cancer	97 (17)		340 (23)		
Colorectal cancer	23 (4)		90 (6)		
**Chemotherapy**					
No	264 (46)		906 (66)		< 0.001
Yes	308 (54)		457 (34)		
**Comorbidities**					
No	209 (42)		95 (39)		0.455
Yes	293 (58)		150 (61)		
**Anxiety and depression**, (**HADS)**					
Depression, (0-21)	561	3.4 ± 3.2	275	3.0 ± 3.2	0.122
Anxiety, (0-21)	561	5.5 ± 4.4	275	4.3 ± 3.7	< 0.001
**Cancer-related fatigue, (MFI)**					
General Fatigue, (4-20)	544	11.4 ± 4.5	269	10.4 ± 4.3	0.004
Physical Fatigue, (4-20)	547	11.2 ± 4.3	270	10.6 ± 4.3	0.050
Reduced Activity, (4-20)	545	10.6 ± 4.1	268	9.6 ± 3.8	< 0.001
Reduced Motivation, (4-20)	547	8.7 ± 3.5	271	8.5 ± 3.4	0.326
Mental Fatigue, (4-20)	545	9.3 ± 4.1	271	8.0 ± 3.6	< 0.001
**Exercise stage, (ESAI) endurance training**					
Not physically active (Stages 1–3)	302 (62)		99 (47)		< 0.001
Physically active (Stages 5–4)	187 (38)		112 (53)		
**Exercise stage, (ESAI) resistance training**					
Not physically active (Stages 1–3)	371 (78)		129 (68)		0.007
Physically active (Stages 4–5)	103 (22)		60 (32)		
Outcome expectations for exercise (NRS)					
Health, (0-10)	553	9.3 ± 1.4	271	8.5 ± 2.5	< 0.001
Symptoms, (0-10)	553	7.5 ± 2.3	271	6.5 ± 3.0	< 0.001
Cancer recurrence, (0-10)	553	5.7 ± 2.8	271	5.3 ± 3.0	0.052
**Exercise self-efficacy (EBSS, 0–90)**	544	49.9 ± 16.2	154	45.0 ± 20.1	< 0.001
**Kinesiophobia (TSK-SV-14, 14-56)**	500	23.2 ± 5.1	233	24.5 ± 5.0	0.001

^a^Percentage is based on available data for each variable.

*HADS* Hospital Anxiety and Depression Scale, *MFI* Multidimensional Fatigue Inventory, *ESAI* Exercise Stage Assessment Instrument, *EBSS* Exercise Barriers Self-efficacy Scale, *m-TSK-SV-14* modified Tampa Scale for Kinesiophobia.

### Step 2: Completers vs. drop-outs

In total, 167 out of 577 participants (29%) dropped out from the intervention. Reasons for withdrawal are reported in Table 3; the most common reason (21%) was that the intervention program was too time-consuming. A greater proportion of completers were classified as “physically active” (i.e., the “action” or “maintenance” stages according to the ESAI questionnaire) regarding endurance training at baseline, and completers had less kinesiophobia than drop-outs (Table 4). No other differences were detected between completers and drop-outs.

**Table 3:** Reasons for dropping out from the exercise intervention

Reasons for dropping out	*n*	%
Too time-consuming	35	21
Side-effects from treatment	23	14
Stress	14	8
No motivation	8	5
Other illness	7	4
Did not like the training	7	4
Other^a^	15	9
No reason given	58	35
Total	167	

^a^For example, exercise too exhausting, death in family, and psychological distress.

**Table 4:** Comparison of completers vs drop-outs in the exercise trial, based on baseline data. *N* vary due to missing data

	Completers		Drop-outs		
	*n* (%)*	Mean ± SD	*n* (%)	Mean ± SD	*P-value*
**Age**, years	410	59 ± 12	167	58 ± 13	0.428
**Sex**					
Men	83 (20)		29 (17)		0.428
Women	327 (80)		138 (83)		
**Living situation**					
Living with partner	315 (79)		116 (75)		0.273
Living without partner	83 (21)		39 (25)		
**Education level**					
University	250 (62)		86 (55)		0.101
Not University	151 (38)		71 (45)		
**Diagnosis**					
Breast cancer	321 (78)		136 (82)		0.624
Prostate cancer	71 (17)		26 (15)		
Colorectal cancer	18 (4)		5 (3)		
**Chemotherapy**					
No	183 (45)		77 (46)		0.747
Yes	227 (55)		90 (54)		
**Comorbidities**					
No	157 (43)		52 (38)		0.269
Yes	207 (57)		86 (62)		
**Anxiety and depression, (HADS)**					
Depression, (0-21)	404	3.2 ± 3.0	157	3.8 ± 3.6	0.053
Anxiety, (0-21)	404	5.4 ± 4.3	157	5.9 ± 4.6	0.189
**Cancer-related fatigue, (MFI)**					
General fatigue, (4-20)	394	11.3 ± 4.4	150	11.6 ± 4.6	0.583
Physical fatigue, (4-20)	400	11.1 ± 4.3	147	11.5 ± 4.2	0.281
Reduced activity, (4-20)	397	10.6 ± 3.9	148	10.7 ± 4.4	0.935
Reduced motivation, (4-20)	400	8.6 ± 3.4	147	8.9 ± 3.7	0.461
Mental fatigue, (4-20)	396	9.2 ± 3.9	149	9.6 ± 4.4	0.331
**Exercise stage, (ESAI) endurance training**					
Not physically active (Stages 1–3)	207 (58)		95 (71)		0.007
Physically active (Stages 4–5)	149 (42)		38 (29)		
**Exercise stage, (ESAI) resistance training**					
Not physically active (Stages 1–3)	265 (77)		106 (81)		0.388
Physically active (Stages 4–5)	78 (23)		25 (19)		
**Outcome expectations for exercise (NRS)**					
Health, (0-10)	402	9.3 ± 1.3	151	9.3 ± 1.4	0.714
Symptoms, (0-10)	402	7.6 ± 2.1	151	7.3 ± 2.6	0.213
Cancer recurrence, (0-10)	402	5.9 ± 2.7	151	5.4 ± 3.1	0.093
**Exercise self-efficacy, (EBSS, 0–90)**	394	50.5 ± 15.6	150	48.1 ± 17.6	0.117
Kinesiophobia, (TSK-SV-14, 14-56)	364	22.9 ± 5.0	136	24.0 ± 5.3	0.044
**Physical activity and cardiorespiratory fitness level**					
MVPA, h/day	375	1.3 ± 0.8	143	1.2 ± 0.7	0.061
VO_2_max, ml/kg/min	343	30.7 ± 7.1	122	29.4 ± 6.8	0.124

^a^Percentage is based on available data for each variable.

*HADS* Hospital Anxiety and Depression Scale, *MFI* Multidimensional Fatigue Inventory, *ESAI* Exercise Stage Assessment Instrument, *EBSS* Exercise Barriers Self-efficacy Scale, *m-TSK-SV-14* modified Tampa Scale for Kinesiophobia, *MVPA* Moderate-to-vigorous intensity physical activity, *VO*_*2max*_ maximal oxygen uptake.

## Discussion

The present study provides information about differences between participants and decliners, as well as between completers and drop-outs in a 6-month exercise intervention for patients undergoing cancer treatment. We found that a larger proportion of participants in the RCT, compared to decliners, were women, diagnosed with breast cancer, scheduled to undergo chemotherapy treatment, and had a university education. The participants were younger, had higher outcome expectations and exercise self-efficacy, less kinesiophobia, higher levels of anxiety, and more cancer-related fatigue. Moreover, a larger proportion of the participants, compared to decliners, were classified as “not physically active” at baseline. However, within the group who participated, a larger proportion of those who completed the intervention were classified as “physically active” at baseline and reported lower levels of kinesiophobia compared to those who dropped out.

For sociodemographic characteristics, our findings are mainly in line with previous studies investigating differences between participants and decliners. The propensity for older patients to decline participation in exercise interventions has been reported in cancer populations [13, 31, 41], and consequently, they are underrepresented in clinical trials [15, 25]. Higher educational level among exercise trial participants has previously been reported in samples of cancer survivors [18, 40] and other clinical populations [20, 30]. One possible explanation is that higher education is positively associated with health literacy [42], suggesting that those who are more health literate may be more open to advice regarding physical activity and therefore may be more likely to participate in health-enhancing interventions. Consequently, to improve generalizability future research should incorporate methods to identify and overcome specific barriers for participation among older people and those with lower educational levels.

Interestingly, our participants reported higher levels of anxiety and more cancer-related fatigue at baseline compared to decliners. This is in contrast to previous studies where decliners have reported more psychological distress [18] and fatigue [40] than participants in exercise interventions. There are no obvious differences in inclusion/exclusion criteria or intervention between the two previous studies and the present one that may explain these contrasting results. It is possible that the awareness about health benefits of exercise during and after cancer treatment has increased since the time of the earlier studies, and consequently, experiencing anxiety and fatigue may nowadays be an incentive for patients to participate. A greater proportion of our participants, compared to decliners, were scheduled to undergo chemotherapy treatment. This result was driven by relatively more women with breast cancer accepting participation compared with other diagnoses. However, among our completers and drop-outs, there was no difference in proportions of patients undergoing chemotherapy, indicating that treatment regimen is less influential once the exercise intervention has started. Moreover, there were no differences between participants and decliners regarding co-morbidities, which is in line with two previous studies [18, 40] but in contrast with other research [13]. One possible explanation for these disparate findings is that co-morbidity has been assessed with different methods.

For exercise-related characteristics, we observed that a greater proportion of our participants, compared to decliners, were classified as “not physically active” according to the exercise stages in the ESAI scale. This finding is supported by previous research on cancer survivors reporting lower physical activity among trial participants [40] although their study also identified a subgroup of decliners who already exercised habitually and wished to continue exercising on their own. In the present study, we did not have complete data on decliners and reasons for declining were stated by one-third of the 1451 eligible. Among the remaining two-thirds, there may have been subgroups of decliners already physically active and therefore not motivated to participate in a comprehensive RCT. In comparison to decliners, our participants demonstrated higher levels of self-efficacy and outcome expectations of exercise on improved health and symptom reduction. These results correspond well with previous research in populations with breast cancer [40] and mixed cancer diagnoses [18, 40]. It is not surprising that exercise trials with comprehensive interventions attract participants with higher self-efficacy and higher outcome expectations of exercise. To reach patients with lower levels, study designs other than RCT may be used, taking into account patients’ exercise preferences [10] and prioritizing effectiveness rather than efficacy. We also observed that participants reported a slightly lower level of kinesiophobia, a phenomenon less studied in this setting. However, it is important to note that both our participants and decliners reported low levels of kinesiophobia, classified as subclinical and mild kinesiophobia, respectively [27]. Given that our decliners presented mild levels of kinesiophobia, other factors may be more influential in their decision to decline participation in our RCT. However, assessing kinesiophobia among cancer survivors could still be of great value as it could identify those who, for this reason, would hesitate in taking part in physical exercise.

Of those invited, 29% of the targeted population accepted participation in the 6-month randomized controlled exercise trial. Previous exercise intervention targeting patients with cancer have reported recruitment rates varying from 9.5 to 44% [8, 16, 18, 34, 38, 40] where the majority report acceptance rates over 30%. One study with an acceptance rate below 30% [16] identified three main reasons for declining participation: (1) lack of interest in the study, (2) felt too busy to participate, and (3) did not want to travel to the training facility. These results are in line with the findings in the Phys-Can RCT, where travel distance was the major reason for declining participation [9]. Although not stated as a reason in the present study, it is possible that the randomization to two different exercise intensities may have deterred eligible participants as they may have been hesitant to exercise at a non-preferred intensity. In addition, 6 months is a long-time commitment and this may have contributed to lower acceptance rates than reported in previous trials.

In the present study, we also compared those who completed the full 6-month exercise intervention with the 29% who dropped out. Very few differences in sociodemographic, health-, and exercise-related characteristics were observed. We found that those who completed the intervention were more likely to be in a “physically active” exercise stage at baseline. Interestingly, although a greater proportion of completers regarded themselves as “physically active” at baseline, there were no differences in time spent in MVPA or in VO_2max_ between completers and drop-outs. Overestimating the level of physical activity is common in subjective measures [14], which could be the case in the present study. However, it should be noted that the ESAI questionnaire does not evaluate the physical activity level per se, but rather how “ready” one perceives oneself regarding exercise. Perceiving oneself as physically active does not necessarily translate into a greater activity level (MVPA level) or VO_2max_, which could be another reason for the discrepancies found in the present study.

The majority of drop-outs occurred during the introduction period and the most commonly reported reason for leaving was the intervention being too time consuming. Even if there were no differences between groups in exercise self-efficacy, it is possible that being in “action” or “maintenance” stage from start made it easier to prioritize exercise and overcome e.g. time constraints. However, we do not have a complete picture of reasons for drop-out since 35% of participants did not report a reason for leaving the study. Future research in the field of exercise oncology may gain by developing interventions that are individualized and adapted to each patient’s situation and preferences. We also observed that completers had a lower level of kinesiophobia compared to drop-outs; however, overall levels were low in both groups and may not have had a strong influence on the decision to withdraw.

A 29% drop-out rate is higher compared to previous research, where drop-out rates of around 10% have been reported in mixed cancer populations and for different types of exercise interventions [36]. Our intervention stretched for 6 months, which is longer than other studies reporting smaller drop-out rates [13, 18]. The majority of drop-outs exited the study in the introduction phase, and it is possible that our participants at this time point realized the extent of the commitment and re-evaluated their decision to participate. It may also reflect the side-effects of treatment that typically become more significant over time and was reported as one reason for dropping out.

The current study had some limitations. A majority of the participants eligible for the RCT were women with breast cancer, and thus, the results in this paper may not represent other cancer diagnoses. Moreover, only 30% of our decliners completed the extended questionnaire, which limits the generalizability of the current findings. For decliners, the largest proportions of missing data were on self-efficacy, exercise stage, kinesiophobia, and outcome expectations. The missing data can be attributed to decliners not filling in questionnaires completely or sometimes not at all. The strengths of this study include a large and detailed information on sociodemographic, health-, and exercise-related characteristics associated with participation in, and completion of, a 6-month combined resistance and endurance training intervention.

## Conclusion

To increase generalizability, the recruitment procedures used in comprehensive oncology exercise trials should specifically address barriers for participation among men, patients without university education, and older patients. Individualized efforts should be made to enroll patients with low exercise self-efficacy and low outcome expectations of exercise. To retain participants in an ongoing exercise intervention, extra support may be needed for patients with kinesiophobia, and for those lacking health-enhancing exercise habits at baseline.

## Data Availability

De-identified participant data (including data dictionaries) will be shared upon reasonable request for research purposes by contacting the corresponding author.

## Code availability

N/A.
